# Prophylactic Mesh-related Reoperations and Mesh-related Problems During Subsequent Relaparotomies: Long-term Results From the PRIMA Trial

**DOI:** 10.1097/SLA.0000000000006527

**Published:** 2024-09-06

**Authors:** Rudolf van den Berg, Louis Matthijs van den Dop, Lucas Timmermans, Michiel van den Berg, Robert E.G.J.M. Pierik, Willem A.R. Zwaans, Daniel Reim, Steven E. Buijk, Jerome P. van Brussel, Johan F. Lange, Johannes J. Jeekel, Pieter J. Tanis

**Affiliations:** * Department of Surgery, Erasmus University Medical Centre, Rotterdam, The Netherlands; † Department of Surgery, Radboud University Hospital, Nijmegen, The Netherlands; ‡ Department of surgery, Treant Zorggroep, Emmen, The Netherlands; § Department of Surgery, Isala Clinic, Zwolle, The Netherlands; ‖ SolviMáx, Center of Excellence for Abdominal Wall and Groin Pain, Máxima Medisch Centrum, Veldhoven, The Netherlands; ¶ Department of Surgery, Máxima Medical Centre, Veldhoven, The Netherlands; # NUTRIM School of Nutrition and Translational Research in Metabolism, Maastricht University, Maastricht, The Netherlands; ** Department of Surgery, School of Medicine and Health, Technical University of Munich, Munich, Germany; †† Department of Surgery, IJsseland Hospital, Capelle aan den Ijssel, The Netherlands; ‡‡ Department of Vascular Surgery, Franciscus Gasthuis & Vlietland, Rotterdam, The Netherlands; §§ Department of Neuroscience, Erasmus University Medical Center, Rotterdam, The Netherlands

**Keywords:** complications, mesh, onlay, prophylactic, reoperation, retrorectus

## Abstract

**Objective:**

To evaluate all mesh-related problems during reoperations after mesh reinforcement of the abdominal wall 15 years after the start of the PRIMA trial.

**Background:**

Prophylactic mesh reinforcement during closure of a midline laparotomy has proven to reduce the incidence of incisional hernia, especially in high-risk patients, but long-term mesh-related morbidity is largely unknown.

**Methods:**

Patients receiving a prophylactic onlay or retrorectus mesh in the PRIMA trial between 2009 and 2012 were included on an as-treated basis from participating centers that made reoperation notes available. The main outcomes were the incidences of complications requiring mesh explantation, mesh-related ileus, and mesh-related problems during laparotomy for other diagnoses.

**Results:**

Out of 373 patients randomized to prophylactic mesh reinforcement, 242 were included: 127 with onlay and 115 patients with retrorectus mesh. Median follow-up is 27 months (interquartile range: 12–78). Thirty-four patients underwent reoperation for any reason during the entire follow-up, 22 after onlay (17.3%), and 12 after retrorectus mesh (10.4%). The reoperation rate for complications that required mesh explantation was 4/127 (3.1%) after onlay and 0/115 (0%) after retrorectus mesh. Mesh-related ileus occurred in none of the onlay group, and 3/115 (2.6%) in the retrorectus group. During subsequent laparotomies for other primary diagnoses, adhesions to the mesh were noted in 3/10 patients in the onlay group and 1/5 patients in the retrorectus group. Overall, the mesh was removed in 10/127 (7.9%) in the onlay group and 7/115 (6.1%) patients in the retrorectus group.

**Conclusions:**

In high-risk patients receiving a prophylactic mesh during midline laparotomy closure, low incidences of mesh-related complications requiring reoperation and mesh-related problems during unrelated subsequent laparotomies were found, for both the onlay and retrorectus techniques.

One of the most frequent postoperative complications after open abdominal surgery is incisional hernia (IH) formation. Specifically, patients undergoing abdominal aortic aneurysm (AAA) repair or those with obesity are at high risk of developing an IH.^1,2^ IH after midline laparotomy may occur in well over 30 to 60% of these high-risk patients.^3^ Measures to prevent this complication are crucial for enhancing patient care given the patient burden related to this complication, as well as the impact on health care resources and costs.

Prophylactic mesh reinforcement has been demonstrated to significantly lower the incidence of IH in high-risk patients.^4–6^ Besides the additional operative time, implementation of prophylactic mesh reinforcement is probably hampered by fear of mesh-related complications.^7^ This hesitance towards the use of a preventive mesh is based on the use of foreign body material in general, as well as specific complications related to its placement in the abdominal wall.^7,8^

Mesh-specific complications can either be the cause of reoperations or the mesh itself might have an impact on future laparotomies. In the initial postoperative phase, the mesh might become infected, requiring explantation.^9^ During the initial healing process, incomplete closure of abdominal wall layers could result in exposure of the abdominal contents to the mesh. This might lead to adhesion-related small bowel obstruction. Approximately 5% of patients who undergo abdominal surgery are readmitted to the hospital for complications that may be related to these adhesions.^10,11^ Adhesions of the bowel to the mesh can also complicate reentrance to the abdominal cavity with the risk of inadvertent enterotomies.^12–14^ However, little literature is available on mesh-related problems for which a reoperation is required, or problems during future laparotomies for other diagnoses in patients who underwent prophylactic mesh reinforcement of the abdominal wall.

The randomized multicenter PRIMA trial included patients with high risk for IH after midline laparotomy due to a body mass index (BMI) ≥27 or undergoing AAA repair.^1^ Availability of data on reoperations in this trial is necessary to better enable a decision on prophylactic mesh placement during abdominal closure in high-risk patients. Therefore, the aim of the current study was to evaluate patients who underwent prophylactic onlay or retrorectus mesh reinforcement in the PRIMA trial and to determine the incidence of complications requiring mesh explantation, the incidence of mesh-related ileus, and the incidence of other mesh-related problems during long-term follow-up. Furthermore, mesh explantations during subsequent laparotomies for other diagnoses were assessed.

## METHODS

### Study Design and Patient Selection

The methods and study design of the PRIMA trial have been described previously.^1^ In short, patients aged ≥18 years and undergoing elective midline laparotomy with an increased risk of developing IH were included from 11 centers across 3 countries. High risk for IH could either comprise undergoing an open AAA repair or having a BMI of 27 kg/m^2^ or higher. Before surgery, patients were randomized to receive either of 3 closure techniques of the abdominal wall: (1) primary suture, (2) onlay mesh reinforcement, or (3) retrorectus mesh reinforcement. Both the trial’s main findings and long-term primary outcome data have been published.^1,2^

For the current study, all patients who received either an onlay or a retrorectus mesh on an as-treated basis were selected. Centers that originally took part in the PRIMA trial were approached to provide the study team with data on all reoperations during the entire follow-up. Only the centers that were able to provide these data, including full-text operative notes, participated in this present study.

The medical ethics board of the Erasmus University Medical Centre approved the conduction of the PRIMA trial. A waiver for ethical approval was acquired for additional analyses. This subgroup analysis of the PRIMA study was conducted according to the “Strengthening the Reporting of Observational studies in Epidemiology” guidelines.^15^

### Mesh Reinforcement

For the onlay mesh reinforcement group, a lightweight polypropylene mesh (Optilene mesh LP, 6×35 cm; B Braun Surgical SA) was fixed to the anterior rectus fascia with a 3 cm overlap. Fixation was accomplished using 4.0 mL of fibrin sealant (Tisseel; Baxter Healthcare). The edges and the center of the mesh were glued to the tissue and positioned with the back of a pair of forceps on the entire surface. Subcutaneous tissue and skin were closed with sutures according to the surgeons’ preference.

For the retrorectus mesh reinforcement group (referred to as sublay mesh in the prior publications), a posterior plane was established between the rectus muscle and posterior rectus sheath, and caudally to the arcuate line between the rectus muscle and peritoneum. An identical mesh was placed on the sutured posterior rectus fascia, with a 3 cm overlap. The mesh was fixated equally for the onlay mesh reinforcement using fibrin glue. The subcutaneous tissue and skin were closed with sutures according to the preference of the surgeon.

### Data Extraction

In this study, the available study data and relevant reoperation notes were assessed by 2 authors (P.J.T. and R.V.D.B.). The operative reports were assessed using a standardized form that included the following items: signs of infection or abscess in the abdominal wall, whether cultures were taken, presence of an enterocutaneous fistula, visualization of the mesh during reintervention, mesh removal, transsection of the mesh during reentrance of the abdominal cavity, fluid accumulation surrounding the mesh, adhesions to the mesh of intra-abdominal organs, clinical signs of ileus, and technique of closure of the abdominal wall during relaparotomy.

### Primary and Secondary Objectives

The primary objective was to determine the incidence of reoperation for complications requiring mesh explantation, as well as the incidence of mesh-related ileus requiring reoperation, and problems during subsequent laparotomies for other diagnoses. Secondary objectives were to evaluate the type of problems that were encountered as well as the surgical and clinical consequences, and to assess possible differences in the primary outcome between onlay and retrorectus mesh reinforcement. In addition, the number of reoperations for fascial dehiscence or IH in the onlay and retrorectus groups was determined.

### Statistical Analysis

Discrete variables were reported as absolute numbers and percentages, and continuous variables were reported as mean and SD or as median and interquartile range (IQR). Baseline characteristics and outcome parameters were reported separately for the 2 study arms of interest (onlay and retrorectus mesh). Discrete outcomes were analyzed using χ^2^ tests or Fisher exact tests, as appropriate. Continuous variables were compared using a student *T* test or a Wilcoxon sum-rank test as appropriate (ie, normality was assessed graphically in quantile–quantile plot). Statistical analysis was performed with R-studio (R-version: 4.0, Ó 2009–2024 RStudio).^16^ A *P* value <0.05 was considered statistically significant.

## RESULTS

### Patient Selection and Reoperations

**FIGURE 1:**
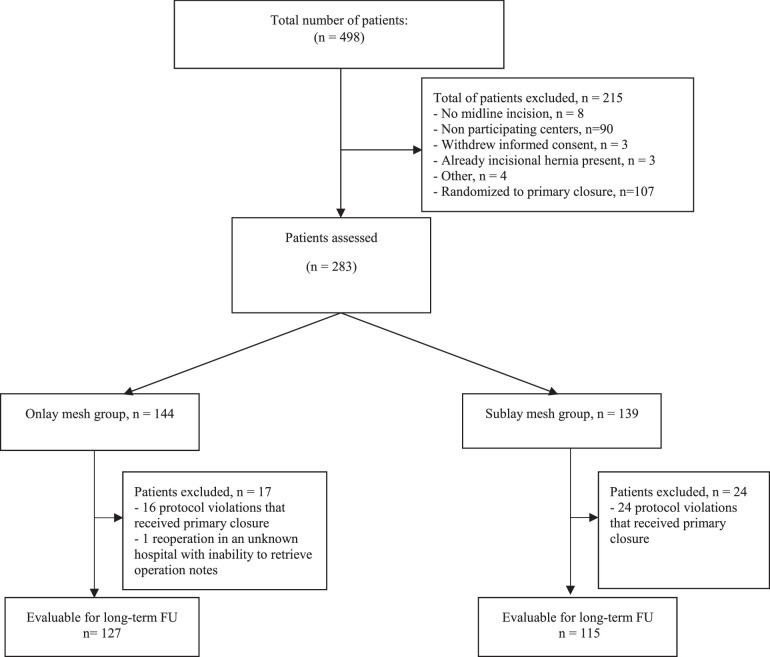
Study flow diagram of the included patients. FU indicates follow-up.

Seven of the 11 hospitals of the original PRIMA trial were able to provide additional information on reoperations and surgical notes. After excluding patients being randomized for primary closure (n = 107) and the 90 patients treated in the other 4, nonparticipating hospitals, 283 patients were assessed for eligibility. Patients in whom no mesh was implanted despite randomization (n = 40 protocol violations) and 1 patient who underwent reoperation in an unknown hospital were excluded. This resulted in a total of 242 patients that could be included in the present analysis (Fig. 1): 127 patients with an onlay mesh and 115 patients with a retrorectus mesh.

The baseline characteristics of the overall study population and the 2 mesh groups are depicted in Table 1. No significant differences in baseline parameters between the onlay and retrorectus mesh groups were found.

**TABLE 1:** Baseline Characteristics of the Study Population

Characteristic	Overall, N = 242*	Onlay, N = 127*	Retrorectus, N = 115*	*P* †
Age, mean (SD)	65 (11)	65 (12)	65 (10)	0.5
Sex				0.7
Male	146 (60)	75 (59)	71 (62)	—
Female	96 (40)	52 (41)	44 (39)	—
BMI, mean (SD)	30.4 (5.9)	30.4 (6.3)	30.3 (5.5)	>0.9
Smoking	155 (73)	84 (74)	71 (71)	0.5
Diabetes	192 (80)	101 (81)	91 (80)	0.8
COPD	209 (87)	107 (86)	101 (89)	0.4
ASA score				0.7
I	25 (10)	16 (13)	10 (9)	—
II	127 (52)	67 (53)	60 (52)	—
III	57 (24)	26 (20)	30 (26)	—
IV	4 (2)	3 (2)	1 (1)	—
Unspecified	29 (12)	15 (12)	14 (12)	—
Previous midline incisions	9 (4)	5 (4)	4 (3)	>0.9
Other hernia (inguinal, umbilical, or epigastric)	32 (14)	17 (14)	15 (13)	>0.9
Type of index surgery for underlying disease				0.8
Vascular	102 (42)	56 (44)	46 (40)	—
Lower GI	70 (29)	36 (28)	34 (30)	—
Upper GI	18 (7)	10 (8)	8 (7.0)	—
HPB	4 (2)	2 (2)	2 (2)	—
Gynecology	44 (18)	20 (16)	24 (21)	—
Urology	4 (2)	3 (2)	1 (1)	—
Steroids				>0.9
No	232 (97)	123 (98)	110 (96)	—
Yes	7 (3)	3 (2)	4 (4)	—
Cytostatics				0.5
No	219 (92)	118 (94)	102 (89)	—
Yes	20 (8)	8 (6)	12 (11)	—

* Mean (SD); n (%).

† Wilcoxon rank-sum test; Fisher exact test.

ASA indicates American Society of Anesthesiologists; COPD, chronic obstructive pulmonary disease; GI, gastrointestinal; HPB, hepato-pancreato-biliary.

### Reoperations

The median follow-up of the 242 patients was 27 months (IQR: 12–78). A total of 34 patients underwent an abdominal reoperation for any reason at any time during follow-up: 22 patients with onlay mesh (17.3%) and 12 patients with retrorectus mesh (10.4%), *P* = 0.12. The median time interval to reoperation was 76 days (IQR: 11–986), which was similar for onlay mesh [76 (IQR: 11–1493)] and retrorectus mesh [80 (IQR: 14–385)]. The total number of reoperations at any time during follow-up for abdominal wall or mesh-related complications was 15 (6.2%): 9 in the onlay group and 6 in the retrorectus group (*P* = 0.54). The total number of relaparotomies for other diagnoses, such as oncological or vascular reasons, during the entire follow-up, was 10 in the onlay group (7.9%) and 5 in the retrorectus group (4.3%), *P* = 0.30. Four patients underwent a laparoscopic reoperation during follow-up.

### Reoperations for Abdominal Wall or Mesh-related Complications

Table 2 provides details regarding the 15 patients who underwent reoperation for abdominal wall (n = 8) or mesh-related complications (n = 7). The mean duration to reoperation was substantially shorter in the onlay group as compared with the retrorectus mesh group, although not reaching statistical significance (21 vs 431 days, *P* = 0.09). All 15 reoperations were performed with a midline incision.

**TABLE 2:** Operation Characteristics of Reoperations for the Abdominal Wall

Characteristic	Overall, N = 15*	Onlay, N = 9*	Retrorectus, N = 6*	*P* †
Time to reoperation‡	197 (370)	21 (23)	431 (490)	0.093
Indication for reoperation				0.12
Hematoma	1 (6.7)	1 (11)	0	—
Ileus	3 (20)	0	3 (50)	—
Infection	2 (13)	2 (22)	0	—
Seroma	1 (6.7)	1 (11)	0	—
Fascial dehiscence	5 (33)	4 (44)	1 (17)	—
IH	3 (20)	1 (11)	2 (33)	—
Fistulas seen during reoperation				>0.9
Yes	1 (6.7)	1 (11)	0	—
No	14 (93)	8 (89)	6 (100)	—
Mesh seen during reoperation				>0.9
Yes	14 (93)	8 (89)	6 (100)	—
Not noted	1 (6.7)	1 (11)	0	—
Mesh removed during reoperation				0.011
Yes	10 (67)	8 (89)	2 (33)	—
No	4 (27)	0	4 (67)	—
Not noted	1 (6.7)	1 (11)	0	—
Cleaving of the mesh during reoperation				>0.9
Yes	4 (27)	2 (22)	2 (33)	—
No	7 (47)	4 (44)	3 (50)	—
Not applicable	4 (27)	3 (33)	1 (17)	—
Fluid collection identified during reoperation				0.089
Yes	4 (27)	4 (44)	0	—
No	10 (67)	4 (44)	6 (100)	—
Not noted	1 (6.7)	1 (11)	0	—
Culturing done during reoperation				0.6
Yes	4 (27)	3 (33)	1 (17)	—
No	11 (73)	6 (67)	5 (83)	—
Adhesions identified during reoperation				>0.9
Yes	7 (47)	4 (44)	3 (50)	—
No	8 (53)	5 (56)	3 (50)	—
Ileus noted during reoperation				0.044
Yes, mesh-related	3 (20)	0	3 (50)	—
Yes, not mesh-related	1 (6.7)	1 (11)	0	—
No	11 (73)	8 (89)	3 (50)	—
Mesh trimming performed during reoperation				>0.9
Yes	2 (13)	1 (11)	1 (17)	—
No	12 (80)	7 (78)	5 (83)	—
Not applicable	1 (6.7)	1 (11)	0	—
Closure type that was performed at reoperation				0.7
New mesh	5 (33.7)	4 (44)	1 (17)	—
PDS loop suture	5 (33)	2 (22)	3 (50)	—
Vicryl suture	3 (20)	1 (11)	2 (22)	—
VAC	1 (6.7)	1 (11)	0	—
Fascia intact after mesh removal	1 (6.7)	1 (11)	0	—

* Mean (SD); n (%).

† Wilcoxon rank-sum test; Fisher exact test.

‡ Time to reoperation in days.

PDS indicates polydioxanone; VAC, vacuum assisted closure.

Four patients with an onlay mesh, but none of the patients with a retrorectus mesh underwent reoperation with mesh explantation for infection, seroma, or hematoma [4/127 (3.1%) vs 0/115 (0%), *P* = 0.055]. One onlay mesh was removed due to seroma (0.8%), one due to abdominal hematoma (0.8%), and 2 due to mesh infection (1.7%). In the onlay group, 4 patients had a fluid collection in the abdominal wall compared with none in the retrorectus group. No abscess formation was seen during any of the reoperations.

Three patients who received a retrorectus mesh underwent reoperation for mesh-related ileus (n = 3/115, 2.6%), whereas none of the patients in the onlay mesh group experienced long-term bowel complications related to the mesh (*P* = 0.11). In one patient with a retrorectus mesh, ileus was caused by adhesions to the mesh. During adhesiolysis, several serosal injuries occurred. The fascia was closed primarily with a Vicryl suture, and the mesh remained in situ. In the 2 other patients, ileus occurred due to herniation of a small bowel loop through the posterior fascia, which became adherent to the lateral side of the mesh. Abdominal wall closure was accomplished by repositioning the mesh and by fixating the mesh with vicryl sutures, after which 2 suction drains were placed. In the other patient, the mesh was refixated with 2 PDS loop sutures.

Five reoperations were performed in the early postoperative period due to fascial dehiscence: 4 patients with an onlay mesh (3,1%) and 1 in the retrorectus mesh group (0.9%). Another 3 patients underwent reoperations for IH later on, of which one was performed in a patient with an onlay mesh (0.8%), and 2 with a retrorectus mesh (1.8%). No significant difference between the reoperation rate for fascial dehiscence or IH between onlay (5/127, 3.9%) and retrorectus mesh (3/115, 2.6%) was found (*P* = 0.56).

During reoperation for abdominal wall complications, the mesh was (partially) removed in 8 of 9 patients with an onlay mesh, and in 2 of 6 patients in the retrorectus mesh group.

### Subsequent Laparotomies for Other Diagnoses

Details of the 15 patients who underwent a subsequent laparotomy for other diagnoses are displayed in Table 3. Surgeons mentioned the mesh in their operative notes in 5 patients with an onlay mesh and 5 patients with a retrorectus mesh. Adhesions to the mesh, but not resulting in ileus, were reported in 3 of 10 patients with an onlay mesh and in 1 of 5 patients with a retrorectus mesh. The mesh was removed during subsequent laparotomies for other diagnoses in 2 and 4 patients, respectively.

**TABLE 3:** Operation Characteristics of Unrelated Relaparotomies

Characteristic	Overall, N = 15	Onlay, N = 10	Retrorectus, N = 5	*P* *
Time to reoperation (d), mean (SD)	632 (727)	910 (746)	77 (145)	0.10
Indication for reoperation				>0.9
Anastomotic leakage	2 (13)	1 (10)	1 (20)	—
Hiatal hernia	1 (7)	1 (10)	0	—
Ileus, not mesh-related	2 (13)	2 (20)	0	—
Oncological	6 (40)	4 (40)	2 (40)	—
Postoperative bleeding	2 (13)	1 (10)	1 (20)	—
Suspected ischemic bowel	2 (7)	1 (10)	1 (20)	—
Incision type that was used during reoperation				0.5
Midline	13 (87)	8 (80)	5 (100)	—
Subcostal	2 (13)	2 (20)	0	—
Abscess	2 (13)	1 (10)	1 (20)	>0.9
Enterocutaneous fistula	1 (6.7)	0	1 (20)	0.3
Mesh identified during reoperation				0.2
No	1 (6.7)	1 (10)	0	—
Yes	10 (67)	5 (50)	5 (100)	—
Not reported	4 (27)	4 (40)	0	—
Fluid collection identified during reoperation				0.2
No	11 (73)	7 (70)	4 (80)	—
Yes	1 (6.7)	0	1 (20)	—
Not reported	3 (20)	3 (30)	0	—
Culturing done during reoperation	2 (13)	2 (20)	0	0.5
Adhesions identified during reoperation				>0.9
No	7 (47)	4 (40)	3 (60)	—
Yes, not directly mesh-related	4 (27)	3 (30)	1 (20)	—
Yes, possibly related to the mesh	4 (27)	3 (30)	1 (20)	—
Mesh trimming performed during reoperation				0.010
Not applicable due to removal	6 (43)	2 (22)	4 (80)	—
No	7 (50)	7 (78)	0	—
Yes	1 (7.1)	0	1 (20)	—
Mesh removed during reoperation	6 (40)	2 (20)	4 (80)	0.089
Type of abdominal wall closure after reoperation				0.9
New mesh	1 (6.7)	0	1 (20)	—
Vicryl suture	3 (20)	2 (20)	1 (20)	—
Prolene suture	1 (6.7)	1 (10)	0	—
PDS suture	9 (60)	6 (60)	3 (60)	—
Single button suture	1 (6.7)	1 (10)	0	—

* Wilcoxon rank-sum test; Fisher exact test.

PDS indicates polydioxanone.

Closure of the abdominal wall after subsequent laparotomies were accomplished using a new mesh in 2 patients in the onlay group. This was a large-size composite mesh (40×15 cm) in a bridging position in one patient and a prolene intraperitoneal mesh in the other patient. The use of layered closure was documented twice in the onlay group.

### Laparoscopic Reoperation

Four patients had a laparoscopic reoperation (3 patients with an onlay and 1 with a retrorectus mesh). No problems directly related to the mesh were mentioned in any of the 4 laparoscopic operations. Two notes mentioned adhesions: (1) between the posterior rectus sheath and the greater omentum in one patient and (2) between the abdominal wall and small intestines in the other. These adhesions were not deemed to be related to the mesh. In one patient, a paramedian entry port was chosen because of suspected adhesions in the midline.

## DISCUSSION

Patients who underwent open AAA repair or had a BMI ≥27 were included in the PRIMA trial on prophylactic mesh reinforcement during closure of a midline laparotomy. This long-term follow-up study with a median follow-up of 27 months, showed that an onlay or retrorectus mesh both have low incidences of mesh-related reoperations or problems during subsequent laparotomies. Small nonsignificant differences between the onlay and retrorectus mesh position were found, but the notion should be made of an underpowered analysis. Reoperation with mesh explantation for abdominal wall complications occurred only after onlay mesh, whereas reoperation for mesh-related ileus was only performed after retrorectus mesh. During subsequent laparotomies for other diagnoses, adhesions to the mesh were noted in both groups. With regard to the effectiveness of mesh reinforcement, the proportion of reoperations for fascial dehiscence or IH was 3.9% after onlay and 2.6% after retrorectus mesh, which is lower than expected. These long-term follow-up data show low incidences of mesh-related reoperations and mesh-related problems during subsequent laparotomies, which should further support the use of prophylactic mesh reinforcement during closure of midline laparotomies in high-risk patients.

A mesh is most often used in abdominal wall closure during elective IH repair surgery. A registry based cohort study in 2016 found that the cumulative incidence of a mesh-related complication requiring surgical treatment was 5.6% at 5 years for an open mesh repair (95% CI: 4.2%–7.0%).^7^ In the present study, 7 patients underwent reoperation for mesh-related complications [7/242 (2.9%)] after prophylactic mesh placement, which seems to be a lower risk as compared with IH repair surgery. This underlines the importance of IH prevention.

Among studies that have been published on prophylactic abdominal mesh reinforcement to prevent IH,^17–37^ only a little information about long-term mesh-related problems is documented. In a trial that randomized 288 patients undergoing open surgery for morbid obesity (144 mesh, 144 no mesh), Pans et al^25^ documented, with a mean follow-up of 30 months, one case with adhesion-related small bowel obstruction requiring laparotomy, during which the intraperitoneal mesh was removed without difficulty. No information is given regarding the role of the mesh in the formation of the adhesion. Therefore, the current study provides useful data for surgeons who fear mesh-related complications during midline closure.

Curro et al^33^ reported no reoperations due to mesh-related complications in 45 obese patients undergoing retromuscular retrorectus mesh reinforcement during biliopancreatic diversion with 55% of patients completing the 12 months follow-up. The prophylactic mesh was explanted in 2 patients who developed IH. Furthermore, 2 additional studies, reported on reoperation. Caro-Tarrago et al^34^ reported the 5-year outcomes of 160 patients randomized to primary closure or onlay prophylactic mesh. No cases of chronic pain, seroma, infection, or hematoma were documented at 5-years. Although they reported significantly more seromas in the mesh group in their initial report, none of these resulted in reoperation.^38^ Five-year reoperation rate for IH was 15% (12/80) in the control group and 1% (1/80) in the experimental, onlay mesh closure, group. Abo-Ryia et al^37^ randomized 64 morbidly obese patients undergoing open bariatric surgery to primary closure and preperitoneal mesh placement, and reported similar early postoperative wound problems that were all managed conservatively. The reoperation rate for IH was 9/32 and 1/32, with 48 and 49 months of follow-up, respectively. In the present study, 8 reoperations for fascial dehiscence or IH were performed in 242 patients after mesh augmentation (3%), matching the previously reported rates.

Another group of patients at high risk of IH are those undergoing midline laparotomy in the emergency setting. Prophylactic mesh reinforcement was retrospectively studied by Bravo-Salva and colleagues in 266 patients who underwent emergency midline laparotomies, of whom 187 had a minimum follow-up of 2 years (131 primary closure, 56 mesh). Chronic mesh infections were diagnosed in 2 out of 56 patients (4.3%),^39^ but no mesh explantations were needed. These are remarkably good outcomes, given our documented 1.6% of prophylactic mesh explantation as a result of mesh infection in the elective setting. However, another study by Kumar et al^40^ described prophylactic mesh reinforcement after laparotomy for intestinal perforation with peritonitis, which resulted in a 47% (7/15) mesh explantation rate. Thus, in our study, the need for explantation of a prophylactic mesh due to infection is generally low. There is even evidence showing the low need for explantation in contaminated or even dirty operative fields, based on a randomized controlled trial studying prophylactic retrorectus nonabsorbable mesh in a contaminated field in 100 patients.^41^ Wound seroma was reported in 7 patients; however, these seromas did not impair wound healing in any patient. A deep infection occurred in one patient in the mesh group, necessitating mesh removal. No IH repair operations were performed.

Because 2 types of mesh-reinforcement techniques were used in the PRIMA trial, we analyzed the outcomes separately for the 2 mesh locations. From a theoretical point of view, an onlay mesh is more at risk for explantation, because it is located in the subcutaneous tissue. Previously a significantly higher infectious complication rate was reported in the onlay mesh group of the PRIMA trial.^9^ However, the present study showed that the explantation rate for abdominal wall complications is relatively low and not significantly different from the retrorectus technique. In contrast, mesh-related ileus that required laparotomy was only observed in patients who underwent a retromuscular mesh reinforcement. Theoretically, this can only occur when there is a dehiscence or shearing of the posterior fascia, after which the small bowel can adhere to the mesh or even herniate into the rectus muscle compartment along the lateral side of the mesh. This might be prevented by secure closure of the posterior fascia using the small-bites closure technique,^42^ which was unfortunately not routine care at the time the PRIMA trial was conducted.

Adhesions are a notorious problem during laparotomy in patients who had prior laparotomies but are feared even more after mesh implantation. When prosthetic material is exposed to the peritoneal cavity, it gives rise to an inflammatory reaction, possibly resulting in adhesions between the mesh and abdominal viscera. This may cause severe complications such as ileus, fistula formation, and subsequent enterotomies during reexploration. The positioning of the mesh could represent a relevant factor in preventing adhesion-related problems, in which ideally the mesh is covered by closed peritoneum.^43^ Remarkably, adhesions to an onlay mesh were found in 3 patients. This probably occurred after fascial dehiscence with subsequent contact of the small bowel to the onlay mesh.

Although the individual responses can vary, both human and animal studies provide evidence that the inflammatory activity related to the mesh mostly depends on the amount of material and its textile structure.^44,45^ The majority of inflammatory problems are associated with small-pore polypropylene meshes. The onlay and retrorectus meshes used in the PRIMA trial were lightweight meshes with large pores. Considering the reduced amount of foreign body, there is less risk of excessive scar formation, and elasticity is maintained. This is supported by the present study. During laparotomies for other diagnoses, only a few problems arising from the presence of a synthetic mesh in the abdominal wall were reported in the operative notes. In a large proportion of operative notes, the mesh was not mentioned at all, which indicates proper integration of the mesh into the abdominal wall, thereby not interfering with subsequent abdominal surgery.

Recently, we published the long-term data regarding the IH rate of the PRIMA trial. This confirmed the effectiveness of mesh reinforcement, but there was still a 24.7% IH rate after onlay mesh and a 29.8% IH rate after retrorectus mesh when analyzed according to the intention to treat principle.^2^ In the present analyses, we excluded patients who did not receive a prophylactic mesh according to randomization, and we only focused on patients undergoing reoperation. In this as-treated population, the reoperation rate for fascial dehiscence or IH was low (overall 8/242), without a significant difference between the onlay and retrorectus techniques. One could argue that there is a trade-off between accepting an additional risk of hernia repair by not using a mesh or an additional risk of mesh-related reoperations. However, the overall 15/242 (6.2%) reoperation rate for abdominal wall or mesh-related complications in the present study is still lower than reported reoperation rates for IH between 15 and 28% after primary suturing.^34,37^

A retrospective analysis of 76 emergency midline laparotomies with a prophylactic mesh found that at 64 months of follow-up, 14.3% of patients with a prophylactic mesh developed an IH, which resulted in 2 reoperations (2.6%).^39^ This is in line with the present study, showing that only a minority of patients undergo reoperation for IH. However, this does not mean that the remaining IHs were not clinically relevant, while patients might be symptomatic but do not want to undergo surgery or have a high operative risk due to comorbidities and physical conditions.

Regarding implications for daily practice, the low risk of mesh-related reoperations combined with the substantially reduced risk of IH formation supports the implementation of prophylactic mesh reinforcement in high-risk patients. We recommend surgeons who consider prophylactic mesh placement after midline laparotomy in patients with an estimated high risk of IH to either ask an experienced abdominal wall surgeon to wash in and close the abdominal wall or that such a colleague will give some training and proctoring in the use of a retrorectus prophylactic mesh combined with small-bites fascial closure.

### Limitations

This study has several limitations. The initial study was not powered to assess the incidence of complications during reoperation after mesh implantation between the two experimental study arms; therefore, nonsignificant outcomes could possibly be explained by relatively low numbers. However, due to a low frequency of reoperation, adequate statistical power would be hard to achieve. Another limitation is that reoperation notes could not be assessed for 115 patients due to not being able to come into contact with the center or unwillingness to participate in this long-term follow-up study. This could lead to potential bias if the population in these centers were different from those analyzed. Finally, mesh augmentation, as performed in the PRIMA trial, was probably not optimal, by not combining it with the small-bites technique, using a lightweight mesh, and execution by nonabdominal wall surgeons.

## CONCLUSIONS

The use of prophylactic mesh in high-risk patients undergoing midline laparotomy to prevent IH seems safe. Mesh explantation rates, mesh-related reoperation rates, and mesh-related difficulties experienced during subsequent abdominal surgery were low. The use of an onlay and retrorectus mesh seems related to different complications, but no significant differences were found due to low incidences.
